# Learning Curve of Endoscopic Lumbar Discectomy – A Systematic Review and Meta-Analysis of Individual Participant and Aggregated Data

**DOI:** 10.1177/21925682241289901

**Published:** 2024-10-01

**Authors:** Chan Hee Koh, James Booker, David Choi, Danyal Zaman Khan, Hugo Layard Horsfall, Parag Sayal, Hani J Marcus, George Prezerakos

**Affiliations:** 1 Queen Square Institute of Neurology, 61554 University College London, London, UK; 2 Neurosciences Department, 591481 Cleveland Clinic London, London, UK; 3 Department of Neurosurgery, 98546 National Hospital for Neurology and Neurosurgery, London, UK; 4 Wellcome/EPSRC Centre for Interventional and Surgical Sciences, 61554 University College London, London, UK

**Keywords:** lumbar, degenerative disc disease, degenerative, disc, disc herniation, discectomy, endoscopy, minimally invasive

## Abstract

**Study Design:**

A systematic review and meta-analysis of individual participant and aggregated data.

**Objectives:**

To define the learning curves of endoscopic discectomies using unified statistical methodologies.

**Methods:**

Searches returned 913 records, with 118 full-text articles screened. Studies of endoscopic lumbar spine surgery reporting outcomes by case order were included. Mixed-effects nonlinear, logistic, and beta meta-regressions prdwere conducted to define the learning curves.

**Results:**

13 studies involving 864 patients among 15 surgeons were included in total. For transforaminal endoscopic discectomy, the estimated operating time for the first case was 95 min [CI: 87-104], and the estimated plateau was 66 minutes [CI: 51-81]. An estimated 21 cases [CI: 18-25] were required to overcome 80% of this deficit, but near-plateau performance was expected only after 59 cases [CI: 51-70]. The estimated risk of surgical complications on the first case was 25% [CI: 11%-46%], with an 80% reduction in relative risk requiring an estimated 41 cases. The expected postoperative VAS leg pain score after the first case was 2.7 [CI: 1.8-3.8], with an 80% improvement requiring an estimated 96 cases. Similar numbers were required to overcome the learning curves in interlaminar and biportal endoscopic discectomies.

**Conclusions:**

Approximately 60 cases are required to achieve proficiency in endoscopic lumbar spine surgery, although the greatest part of the learning curve can be overcome with 20 cases. This should be considered when designing implementation programmes for surgeons and service providers that wish to incorporate endoscopic spinal surgery into their practice.

## Introduction

Learning curves are an inescapable feature of surgical care. Surgical case volumes are widely recognised to be associated with surgical outcomes, and surgeons are almost invariably slower in the earliest cases of newly-learned operations.^1-4^ This has implications for patient safety, surgical training, and service delivery. Its importance in surgical research and development is also recognised, with recommendations to incorporate learning curves in the design of surgical innovation studies.^5^ Despite its widely recognised importance, the lack of established standards or methodologies in learning curves analyses have resulted in marked heterogeneity within the literature.^6,7^

Endoscopic spinal surgeries are effective for many degenerative lumbar spine diseases,^8^ a group of conditions with an estimated 266 million new diagnoses worldwide each year.^9,10^ While endoscopic spinal surgery offers many advantages over traditional open surgery (including smaller incisions, less postoperative pain, and shorter hospital stay), these procedures are recognised to have difficult learning curves. The spine can be approached in endoscopic procedures via the transforaminal or interlaminar approaches, with the choice depending on surgeon preference, the level of pathology, and the disc’s morphology and anatomical location. A far-lateral disc may be preferably approached via the transforaminal approach and where lesser neural retraction is desired, whereas interlaminar approaches may be preferred in central discal pathology, in other central pathologies such as flaval or facetal hypertrophy, or at L5/S1 where pelvic may obstruct the approach to the neural foramina.^11^ Biportal techniques are alternatives to uniportal techniques which utilise 2 separate channels for optics and instruments, and has the advantage of a lesser barrier to access and more familiar to spinal surgeons with the ability to use standard spinal instruments.^12,13^

There are unique technical challenges posed by endoscopic surgery, including approach under fluoroscopic guidance rather than direct vision, unfamiliarity with the visual appearance of structures in endoscopic surgery, unfamiliarity with instruments and camera, and operating with 2-dimensional visualisation.^14^ This, in addition to limited training opportunities and high set-up costs, have resulted in very limited uptake of endoscopic spinal surgery in many countries, with many fully certified spinal surgeons being unfamiliar with these techniques.

There are several recently published surgical series that utilise disparate methodologies to describe learning curves associated with the uptake of endoscopic lumbar spine surgery. However, there have been no attempts, to our knowledge, to form a unified quantitative characterisation of these learning curves from the many single surgeon series in the literature. Attempts at synthesis of primary studies of surgical learning curves, in both endoscopic spinal surgery specifically and wider surgical learning curve literature generally, have defaulted to mere establishment of the presence of learning curves (eg, early vs late group comparison), qualitative or quantitative descriptive overviews of learning curves, or synthesis of a single parameter of the learning curves that are defined heterogeneously in the primary studies, as opposed to a unified case-performance curve.^15-27^

We therefore undertook meta-regression analyses of individual participant data and aggregated data. This allows investigation of participant-level variables, most pertinently the case-number, to establish learning curves to describe the relationship between experience and performance.

## Materials and Methods

### Study Reporting and Conduct

The protocol was registered with PROSPERO (CRD ***[*** 42023405692 ***]***). The only deviation from the a priori protocol was to establish that meta-analyses will exclude series comprising more than 1 surgeon, rather than making an assumption of even distribution of cases among multiple surgeons.

Ethical approval was not required for this systematic review.

This study is reported in accordance with the PRISMA statement.^28^

### Search Strategy and Selection Criteria

Searches of PubMed and EMBASE were conducted on 10 March 2023, with no limits on date. A broad search strategy was created: (endoscop* AND (spin* OR disc* OR disk* OR decompress* or laminec*) AND (“learning curve” OR “training curve”). Study selection was performed in duplicate by 2 authors independently.

Inclusion criteria were studies that included any measure of operative performance (such as operating time) or outcome (such as patient reported outcomes) in endoscopic spine surgery for degenerative lumbar spine stratified by case number (either given as individual participant data or pooled into case-sequence groups). Studies were excluded if the number of surgeons making up the series was either more than 1 or otherwise not explicitly made clear. Anterior lumbar spine surgery was excluded. Microendoscopic techniques were excluded. Conference abstracts and non-English articles were excluded due to resource constraints.

### Data Extraction

Information on the approach (transforaminal, interlaminar), mode (fully endoscopic, biportal endoscopic), the type of procedure (laminectomy, discectomy, transforaminal/posterior lumbar interbody fusion) were recorded. Grade of surgeons, number of surgeons contributing to the series, and previous fully endoscopic experience as reported in the paper was also recorded. Information on surgical performance or outcomes were extracted. Graphical data were extracted using WebPlotDigitizer.^29^ Primary outcome was operating time, which was found during study preparation to be the most commonly reported at individual participant levels, and also allowed for the computation of the asymptote for plateau performance (see Supplementary Methods). The primary outcome was extracted in duplicate by 2 authors independently. Surgical complications and Visual Analogue Scale (VAS) for leg pain were secondary outcomes, with no other outcomes being found in sufficient number of studies to allow further analysis.

Study quality was assessed using National Institutes of Health case series quality assessment tool.^30^ This was adapted for the purposes of this meta-analysis, with questions regarding statistical methods and follow-up excluded as they were not relevant to this meta-analysis, where all data are re-analysed and follow up is not required. The study summaries and quality assessment in presented in Supplementary Materials (Table 1 and 2).

### Meta-Analysis

Statistical analyses were conducted using *R* statistical programming,^31^ with packages *nlme* for simple linear and nonlinear mixed-effects regressions,^32^ *MASS* for generalised linear mixed-effects regression,^33^ and *glmmTMB* for mixed-effects beta regressions.^34^ Data were visualised using *ggplot2*^35^ and *patchwork*.^36^

For the learning curve analysis, performance or outcome measures were plotted against the case numbers. For the primary outcome, individual participant data were included for analysis. For all other outcomes, several papers reported pooled data with arbitrary groupings (eg, early vs late cases). For aggregated data, the proportions within the aggregated data grouping (eg, early vs late) were weighted by the total number of cases in that case-order grouping. The case number (within the time series) assigned as the midpoint of that group, and then combined in meta-analysis. Outcomes were analysed if they were reported by more than 3 surgeon series.

Operating time are continuous variables, and was therefore analysed by fitting an exponential decay curves (which produce coefficients for learning rate, asymptote, and learning curve gap) using nonlinear mixed-effects metaregression. Complications are binary data points, and therefore necessitated logistic mixed-effect regressions, producing coefficients for expected rate at first case and learning rate, but not for the asymptote as the curve naturally tends towards zero. VAS scores are proportions which necessitated beta mixed-effect regressions, which is similar to logistic regression producing coefficients for expected proportion at first case and learning rate, but not for the asymptote. Further details are available in Supplementary Methods.

## Results

### Study Selection and Characteristics

The search returned 913 records, and 118 full-text articles were screened. 13 studies with 15 series involving 864 patients were deemed eligible for inclusion (Supplementary Figure 1).^37-49^ Study summaries are presented in Supplementary Table 1. All studies reported that the surgeons were fully qualified with previous experience in open spinal operations.

The summary of learning curves for all procedures and outcome variables are listed in Table 1, with the details written below.

**Table 1.** Summary of Learning Curves in Transforaminal Endoscopic Discectomy, Interlaminar Endoscopic Discectomy, and Biportal Endoscopic Discectomy, by Operating Time, Complications, and Postoperative Leg Pain.

	Transforaminal Endoscopic Lumbar Discectomy		Interlaminar Endoscopic Lumbar Discectomy		Biportal Endoscopic Lumbar Discectomy	
Expected number of cases to overcome 80%^a^	Operating time:	21.4 cases	Operating time:	23.1 cases	Operating time:	20.9 cases
	Complications:	41.8 cases	Complications:	70.8 cases	Complications:	43.4 cases
	Postoperative VAS leg pain:	96.8 cases				
Predicted number of cases for 90% of surgeons to overcome 80%^b^	Operating time:	26.7 cases				
	Complications:	71.8 cases				
	Postoperative VAS leg pain:	193.4 cases				
Expected number of cases to plateau^c^	Operating time:	59.3 cases	Operating time:	64.1 cases	Operating time:	57.8 cases
Predicted number of cases for 90% of surgeons to plateau^c^	Operating time:	74.6 cases				

^a^No included studies reporting postoperative VAS leg pain scores.

^b^Unable to produce surgeon-level predictions for interlaminar and biportal endoscopic discectomy.

^c^Logistic regressions for complications and beta regressions for VAS scores do not fit asymptotes, therefore no estimate available for cases to plateau.

Quality assessment suggested that the quality of included studies was high for the purposes of this study, with a mean score of 6.2/7 (Supplementary Table 2). Supplementary Results show no evidence of publication bias.

### Transforaminal Endoscopic Lumbar Discectomy – Operating Time

There were 7 series in 6 studies with 302 patients reporting operating times in transforaminal endoscopic discectomies.^37,40,41,45,47,49^

**Figure 1.**
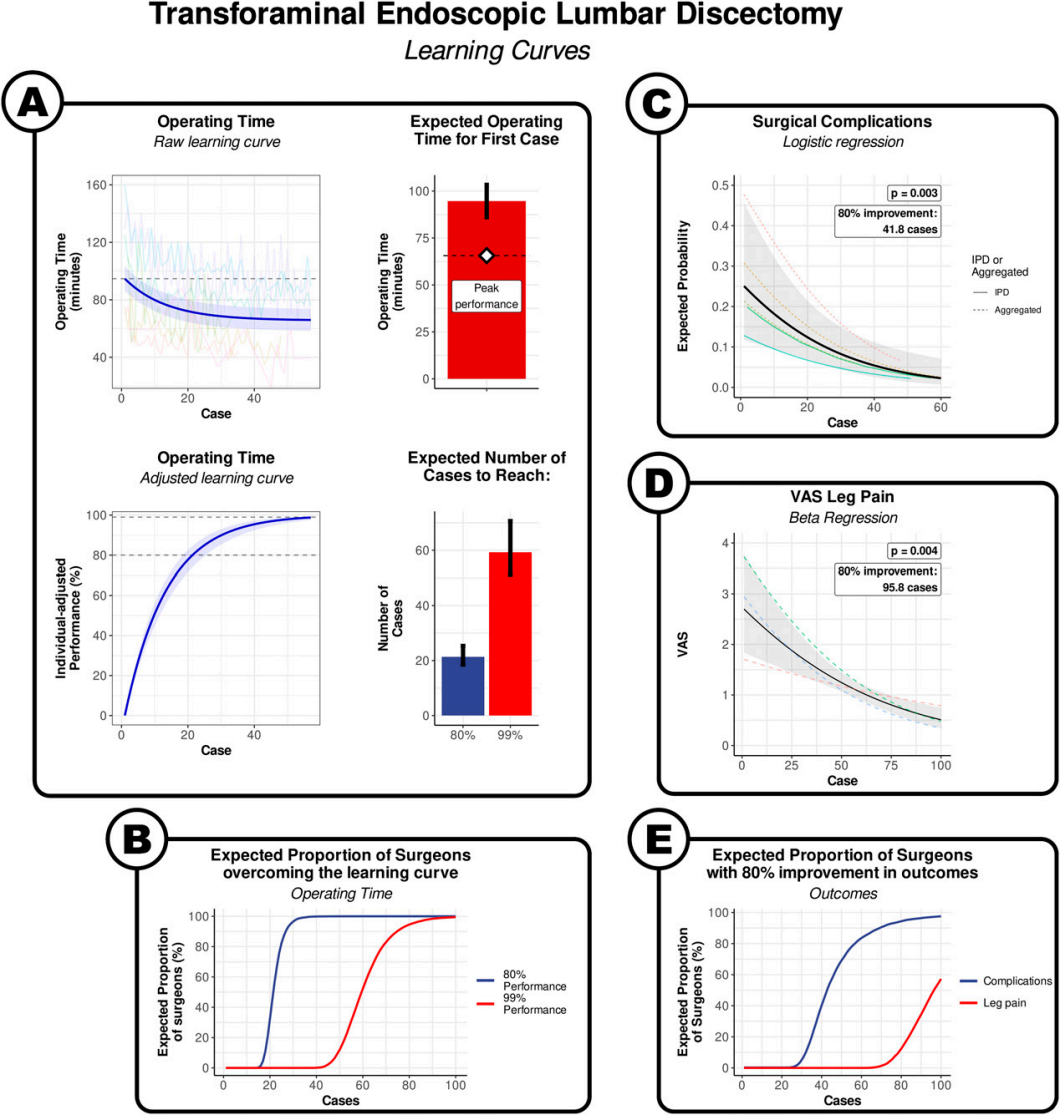
Learning curve of transforaminal endoscopic lumbar discectomy. (A) By operating time. Visualisation of the results of the non-linear meta-regression. The blue line indicates the fitted exponential curve, with the shaded area around the curve indicating the 95% CI. The coloured lines show the individual points from each surgeon series. The horizontal dashed line is the estimate of the time taken in the first case. The bars denote the expected values for first case, and the number of cases to overcome 80% and 99% of the learning curve. The error bars denote the 95% confidence interval. (B) The expected proportion of surgeons to have overcome 80% of the learning curve (blue line) or 99% of the learning curve (red line) by operating time. (C) By surgical complications. Visualisation of the results of logistic regression. The solid black line denotes the average curve between all surgeons, with the shaded grey area indicating the 95% CI. The coloured lines indicate the estimated curves of each individual surgeon series. (D) By postoperative VAS leg pain score. Visualisation of the results of logistic regression. The solid black line denotes the average curve between all surgeons, with the shaded grey area indicating the 95% CI. The coloured lines indicate the estimated curves of each individual surgeon series. (E) The expected proportion of surgeons to have 80% relative risk reduction in surgical complications (blue line), and 80% improvement in postoperative VAS leg pain score (red line).

There was a significant relationship between the case number and operating time (*P* < .0001). This relationship was highly nonlinear and best described by an exponential decay curve (Figure 1A; Supplementary Figure 2), which produced a significantly better fit than a simple linear model on AIC and log-likelihood (*P* < .0001).

The model indicates that the expected operating time for the first transforaminal discectomy was 94.6 min [95% CI: 86.6 to 103.7], and the expected plateau performance was 65.6 min [95% CI: 50.6 to 80.6] (Figure 1A). The estimated average number of cases to overcome 80% of the learning curve was 21.4 [95% CI: 18.4 to 25.0], and a predicted 90% of surgeons could be expected to achieve this mark by their 27th case. We considered that the curve was sufficiently flat at 99% to consider the performance had plateaued, which required approximately 59.3 cases [95% CI: 50.9 to 69.8], and a predicted 90% of surgeons could be expected to achieve this mark by their 75 h case (Figure 1A and B).

### Transforaminal Endoscopic Lumbar Discectomy – Outcomes

26 surgical complications in were reported in 5 studies with 5 series with 201 patients. 2 series presented individual participant data,^43,49^ and 3 series reported pooled data grouped arbitrarily into groups (eg, early vs late).^37,44,50^ The most frequently described complications were dural injury and nerve root injury, with less frequent descriptions relating to surgical technique or adequacy, headaches, haematoma, and infections.

There was a significant association between the case number and surgical complications on logistic metaregression analysis (*P* = .003; Figure 1B). The fitted logistic curve indicated that the expected risk of a surgical complication in the first transforaminal discectomy was 25.1% [95% CI: 11.4 to 46.4]. The log-odds of the risk reduced by .45 (95% CI: .16 to .74) for every 10 cases. It took an expected 41.8 cases for the risk of surgical complications to reduce by 80% from the first case, and a predicted 90% of surgeons could be expected to achieve this mark by their 72nd case (Figure 1C and E).

Postoperative VAS leg pain scores were reported in 3 series in 3 studies, all of which reported aggregated data.^37,38,44^ The most frequently described complications were dural injury and nerve root injury, with less frequent descriptions relating to surgical technique or adequacy, headaches, haematoma, and infections.

There was a significant association between the case number and surgical complications on logistic metaregression analysis (*P* = .001; Figure 1C). The fitted logistic curve indicated that the expected postoperative VAS leg pain score for the first transforaminal discectomy was 2.7 [95% CI: 1.8 to 3.8]. The logit of VAS reduced by .19 [95% CI: .11 to .28] for every 10 cases. It took an expected 96.8 cases for the postoperative VAS to reduce by 80% from the first case, and a predicted 90% of surgeons could be expected to achieve this mark by their 194th case, albeit with a large uncertainty in this estimate (Figure 1D and E).

### Interlaminar Endoscopic Lumbar Discectomy – Operating Time

There were 2 series in 2 studies with 57 patients reporting the operating times in interlaminar endoscopic discectomies.^44,49^

**Figure 2.**
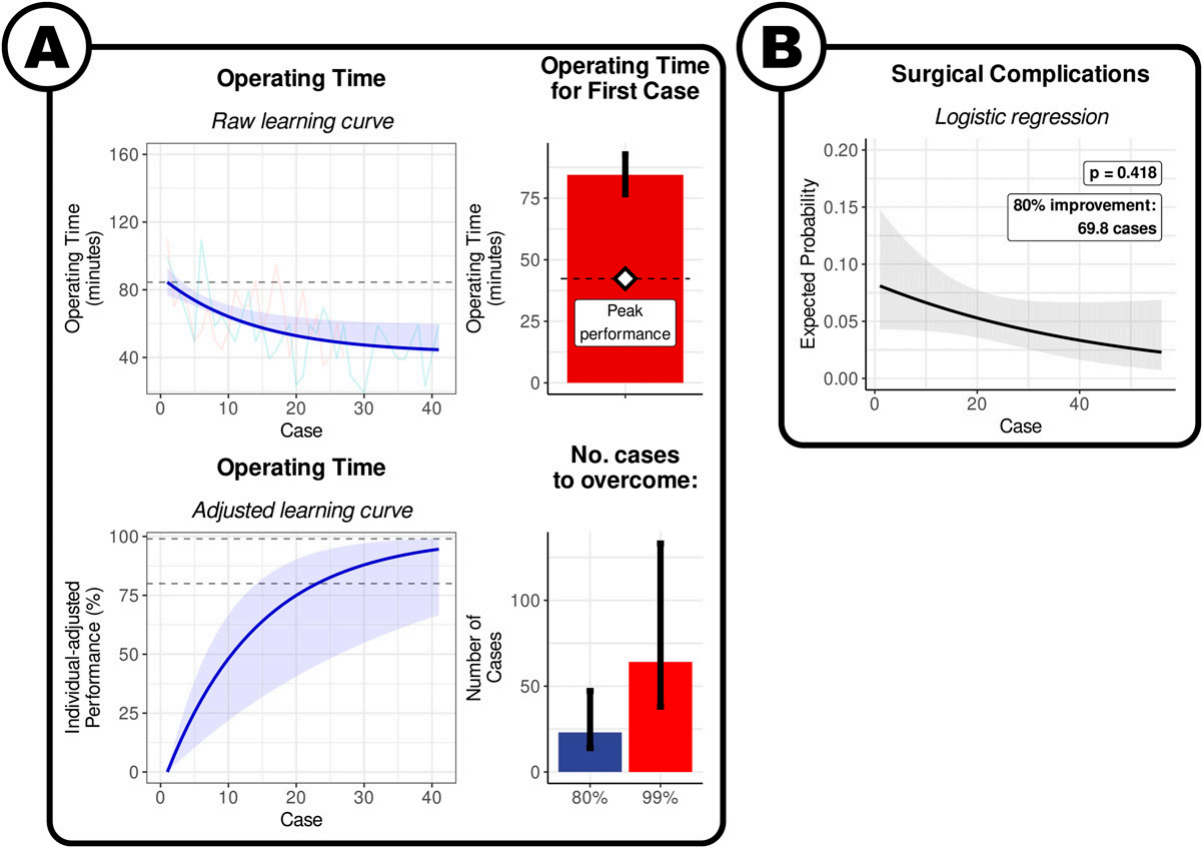
Learning curve of interlaminar endoscopic lumbar discectomy. (A) By operating time. Visualisation of the results of the non-linear meta-regression. The blue line indicates the fitted exponential curve, with the shaded area around the curve indicating the 95% CI. The coloured lines show the individual points from each surgeon series. The horizontal dashed line is the estimate of the time taken in the first case. The bars denote the expected values for first case, and the number of cases to overcome 80% and 99% of the learning curve. The error bars denote the 95% confidence interval. (B) By surgical complications. Visualisation of the results of logistic regression. The solid black line denotes the average curve between all surgeons, with the shaded grey area indicating the 95% CI. The coloured lines indicate the estimated curves of each individual surgeon series.

The model indicates that the expected operating time for the first interlaminar endoscopic discectomy was 84.5 min [95% CI: 76.6 to 92.8], and the expected peak performance once the learning curve has been overcome was 42.3 min [95% CI: 20.6 to 64.0] (Figure 2A). The approximate number of cases to overcome 80% of the learning curve was 23.1 [95% CI: 14.0 to 47.2], and plateau required approximately 64.1 cases [95% CI: 38.1 to 133.1] (Figure 2A). It was not possible to produce a surgeon-level prediction for the number of cases for a given proportion of surgeons to have achieved this mark.

### Interlaminar Endoscopic Lumbar Discectomy – Outcomes

6 surgical complications were reported in 2 studies with 2 series with 117 patients, both of which presented pooled data.^42,49^

The changes in surgical complication rates with increasing experience was not significant on logistic metaregression analysis (*P* = .41; Figure 2B). The fitted logistic curve indicated that the expected risk of a surgical complication in the first interlaminar endoscopic discectomy was 8.1 % [95% CI: 2.2 to 25.4]. The log-odds of the risk reduced by .24 [95% CI: .35 to .83] for every 10 cases. It took an expected 70.8 cases for the risk of surgical complications to reduce by 80% from the first case. It was not possible to produce a surgeon-level prediction for the number of cases for a given proportion of surgeons to have achieved this mark.

There was not enough data to analyse postoperative leg pain for interlaminar endoscopic discectomy.

### Biportal Endoscopic Lumbar Discectomy – Operating Time

There were 2 series in 2 studies with 183 patients reporting the operating times in biportal endoscopic discectomies.^39,48^

**Figure 3.**
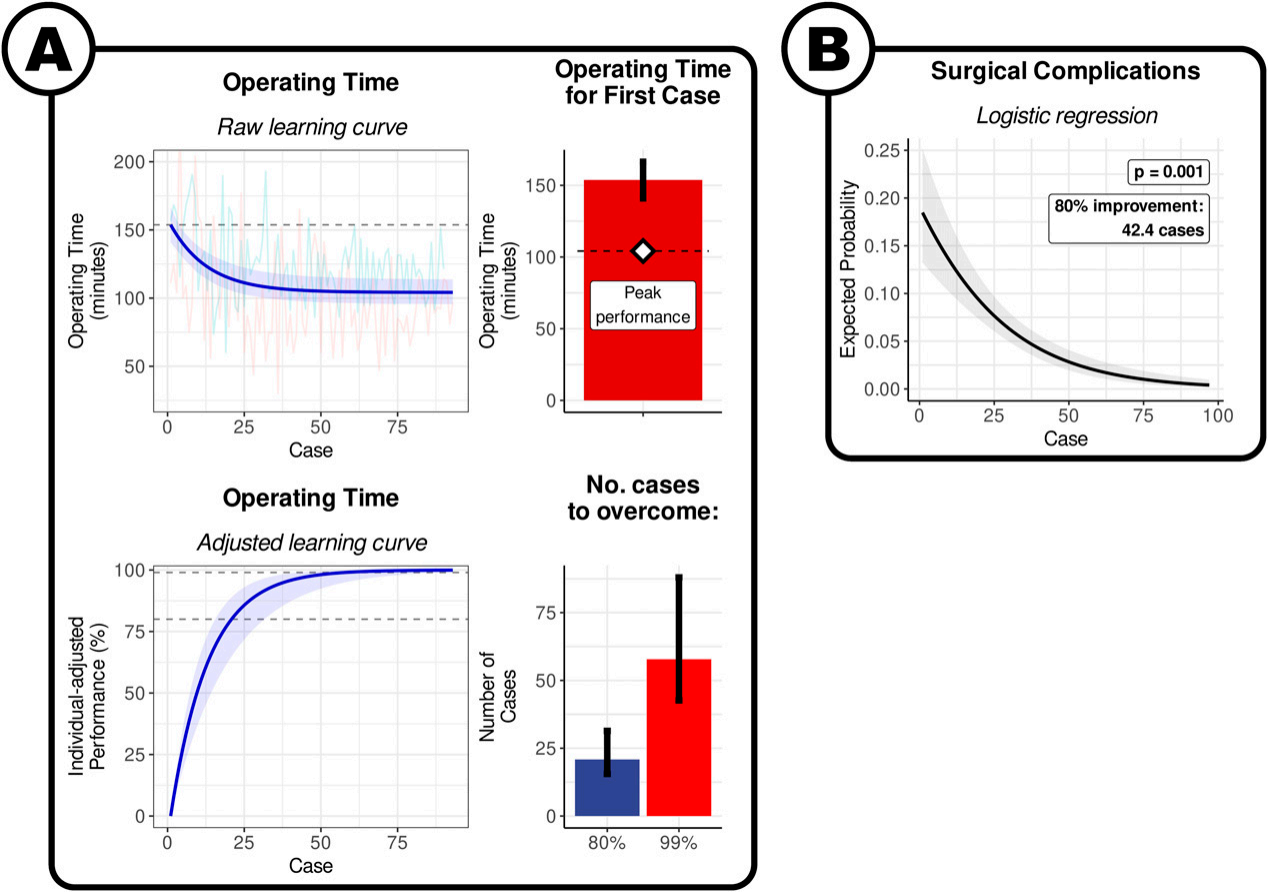
Learning curve of biportal endoscopic lumbar discectomy. (A) By operating time. Visualisation of the results of the non-linear meta-regression. The blue line indicates the fitted exponential curve, with the shaded area around the curve indicating the 95% CI. The coloured lines show the individual points from each surgeon series. The horizontal dashed line is the estimate of the time taken in the first case. The bars denote the expected values for first case, and the number of cases to overcome 80% and 99% of the learning curve. The error bars denote the 95% confidence interval. (B) By surgical complications. Visualisation of the results of logistic regression. The solid black line denotes the average curve between all surgeons, with the shaded grey area indicating the 95% CI. The coloured lines indicate the estimated curves of each individual surgeon series.

The model indicates that the expected operating time for the first biportal endoscopic discectomy was 153.8 min [95% CI: 140.1 to 166.5], and the expected peak performance once the learning curve has been overcome was 86.5 min [95% CI: 104.2 to 121.9] (Figure 3A). The approximate number of cases to overcome 80% of the learning curve was 20.9 [95% CI: 15.5 to 31.4], and plateau required approximately 57.8 cases [95% CI: 42.6 to 88.0]. It was not possible to produce a surgeon-level prediction for the number of cases for a given proportion of surgeons to have achieved this mark.

### Biportal Endoscopic Lumbar Discectomy – Outcomes

9 surgical complications were reported in 2 studies with 2 series with 187 patients, one of which reported individual participant data^48^ while the other reported pooled data.^39^

There was a significant association between the case number and surgical complications on logistic metaregression analysis (*P* = .0008; Figure 3B). The fitted logistic curve indicated that the expected risk of a surgical complication in the first biportal endoscopic discectomy was 18.5% [95% CI: 9.3 to 33.4]. The log-odds of the risk reduced by .42 [95% CI: .18 to .66] for every 10 cases. It took an expected 43.4 cases for the risk of surgical complications to reduce by 80% from the first case. It was not possible to produce a surgeon-level prediction for the number of cases for a given proportion of surgeons to have achieved this mark.

There was not enough data to analyse postoperative leg pain for biportal endoscopic discectomy.

## Discussion

### Summary of Findings

The results of our meta-analysis, involving 864 patients in 15 series presented in 13 studies, estimates that for qualified neurosurgeons and orthopaedic spinal surgeons, around 60 cases are required for surgeons to reach their individual plateau of operating speed for transforaminal, interlaminar, and biportal endoscopic discectomies. The quickest improvement happens in the earliest parts of the learning curve, with 80% of the learning curve being overcome in approximately 20 cases (Figures 1A–3A).

Our analysis suggests that it takes approximately 40 cases for the relative risk of surgical complications in transforaminal and biportal endoscopic discectomy to reduce by 80% from the first case (Figures 1B and 3B). The evidence of uncertain for interlarminar techniques (Figure 2B).

For patient reported outcomes, it required an estimated 95.8 cases for the expected postoperative VAS score for leg pain to reduce by 80% in transforaminal endoscopic discectomy. There were insufficient data to perform the same analyses for interlaminar and biportal techniques.

### Learning Curves in Surgery

Learning curves are an unavoidable aspect of surgical care, with surgeons in the early part of their learning curve being invariably slower and greater risk. The learning curve is a major confounding factor in the evaluation of new surgical procedures or technologies.

The surgical logbook is a long-established method of assessing surgical competence. The indicative numbers of a given procedure are considered a marker of the extent the learning curve has been overcome. However, thresholds are either rarely established, or where established, seldom backed by quantitative evidence or analyses. This paper attempts to address this gap with regards to uptake of endoscopic spinal surgery, which is a procedure that is unfamiliar even to many spinal surgeons.^14,51^

Two recent systematic reviews, a meta-analysis by Ahn et al^15^ and a narrative analysis by Ali et al,^16^ attempted to synthesise the disparate evidence around learning curves in spinal endoscopy. While those studies established the presence of learning curves in spinal endoscopy, they did not produce a description of those learning curves. This study furthers those attempts by formulating mathematical descriptions of the learning curves, with approximately 20 cases required to overcome 80% of the deficit in operating speed, and 60 cases to overcome 99%.

This information has implications for training, supervision, and service implementation of spinal endoscopy. Traditionally, the overcoming and mitigation of surgical learning curves occur with master-apprentice model of surgical training, whereby an experienced surgeon closely supervises trainees for as long as the trainee is deemed not independently competent. This is while managing the extremely delicate balance between maintaining patient safety and the training needs of trainees. A further complication is that training programmes are most often heavily time-based, with a fixed number of years spent in training. In such training programmes, variabilities in case-mix of supervisors, and in the speed of learning of trainees, may allow a sufficient volume in a given technique for a given trainee to reach competence. In more recent times, the industrial and technological capabilities can be leveraged to address the problem of learning curves, such as high surgical simulators and remote video supervision platforms. However, the extent to which these can either replace or augment traditional models of surgical training requires investigation.^52^

### Strengths and Limitations

The strengths of this study lie in pooling the wealth of data available in the relatively large number of included studies to derive an estimate of the learning curve, describing how the expected performance changes with increasing experience. The use of individual participant data for the primary outcome (operating time) allowed the use of nonlinear regression technique using the exponential decay curve describing the plateau performance, the performance gap due to the learning curve, and the learning rate. Performing a meta-analysis also allows the detection of trends that might have been considered statistically insignificant within the smaller datasets of single-surgeon series. Finally, mixed-effects analysis accounts for surgeons possessing inherently different operating speeds, by modelling the asymptote (and the other parameters) as a random effect that can vary between each subject.

The limitations in the literature necessarily produces limitations in this review. Firstly, the patient selection processes, demographics, and definitions of certain outcomes was not always clear, and likely heterogeneous between studies. It was not possible to assess the impact of this as this level of granularity was not available in many of the included studies. Another was the issue of data aggregation in the primary studies which results in an undesirable loss of information, and possibly biases the results of a time-series analysis. We ameliorated this by not pooling aggregated data in the analysis of primary outcomes (which would not be possible to pool and weight in this nonlinear regression analysis in any case). Increased data sharing by future studies, and reporting of individual participant data, will improve the quality of learning curve analyses.

## Conclusions

Approximately 60 cases are required on average to plateau in operating speed in endoscopic discectomy, with 90% of surgeons predicted to have overcome the learning curve of transforaminal endoscopic lumbar discectomy by their 75th case. The quickest improvements happen in the early part of the learning curve, with 80% of speed improvements happening in the first 20 to 25 cases on average, and by their 27th case 90% of surgeons are predicted to have reached this mark in transforaminal endoscopic lumbar discectomy. Depending on the approach, an 80% reduction in the risk of complications requires approximately 40 to 70 cases, and 80% reduction in postoperative leg pain scores after transforaminal discectomy required approximately 96 cases. Training programmes may wish to consider the number of cases, as outlined above, it may take to reach competency in endoscopic lumbar spinal surgery, as well as any surgical procedure in general. Further publications of case series and learning curves in endoscopic lumbar spine surgery are required to produce more precise estimates of learning curves, in particular of interlaminar and biportal techniques, and for greater generalisability of the findings presented.

## Supplemental Material


Supplemental Material - Learning Curve of Endoscopic Lumbar Discectomy – A Systematic Review and Meta-Analysis of Individual Participant and Aggregated Data
Supplemental Material for Learning Curve of Endoscopic Lumbar Discectomy – A Systematic Review and Meta-Analysis of Individual Participant and Aggregated Data by Chan Hee Koh, James Booker, David Choi, Danyal Zaman Khan, Hugo Layard Horsfall, Parag Sayal, Hani J Marcus, and George Prezerakos in Global Spine Journal.
