# Supplementary material for: Learning Curve of Endoscopic Lumbar Discectomy – A Systematic Review and Meta-Analysis of Individual Participant and Aggregated Data
Source: Global Spine J. 2024 Oct 1;15(2):1435–44. doi: 10.1177/21925682241289901 (PMC11559801; doi:10.1177/21925682241289901)
Supplement: Supplemental Material - Learning Curve of Endoscopic Lumbar Discectomy – A Systematic Review and Meta-Analysis of Individual Participant and Aggregated Data [file sj-pdf-1-gsj-10.1177_21925682241289901.pdf]

Supplementary Materials

Table of Contents

Supplementary Methods 2
- Meta-analysis – further details 2
- Publication bias 2

Supplementary Results 3
- Publication bias 3

Supplementary Figures 4

Supplementary Tables 5
- Table 1. Summaries of included studies 5
- Table 2. Quality Assessment 9

Supplementary Methods

Meta-analysis – further details

For the primary outcome, an exponential decay curve was fitted to the time series with nonlinear mixed-effects regression, with autoregressive order of 1 to adjust for temporal correlations in time series data. The random-effects specified as the surgeon that formed a surgical series. The exponential decay was chosen as it gives three parameters: the coefficient ***Asym*** reflects the estimated performance for that surgeon once the learning curve has been overcome; coefficient ***C*** reflects the estimated difference between the performance in the first case and the peak performance; and coefficient ***k*** is the learning curve, with a higher number reflecting quicker learning (**Supplementary Figure 1**). Various models of learning curves were compared using the Akaike Information Criterion (AIC) and log-likelihoods. The population-level confidence intervals were calculated using bootstrap resampling of the model parameters with 10000 replications. The between-surgeon variability and intervals were also calculated to allow estimation of the expected proportion of surgeons to have overcome the learning curve at any given time point.

For the secondary outcomes, general linear mixed-effect meta-regressions were performed with the same random-effects structure with random intercept and random slopes. For dichotomous variables, a logistic meta-regression models were fitted. For continuous data, a simple linear meta-regression was performed as the poor coverage and precision did not allow a reliable non-linear meta-regression. The population-level confidence intervals and prediction intervals were calculated using bootstrapping with 10000 replications.

Publication bias

Given the nature of this individual participant data meta-analysis, it was not possible to conduct a reliable assessment of publication bias using the established methods. The data is not able to be reliably pooled to derive a standard error, as the outcome variable necessarily varies from the others in the same series by the virtue of the place in the series that the operation was performed.

Considering these limitations, we tested a subset of operations that fell between the 18th and 23rd cases on the learning curve using Duval's trim-and-fill analysis and Egger's regression test

Supplementary Results

Publication bias

Testing of operating times of all series between the 18th and 23rd cases showed no evidence of missing studies on Duval’s trim-and-fill analysis, nor significant evidence of publication bias on Egger’s regression test (p=0.43).

Supplementary Figures

Figure 1. Study Flow Diagram

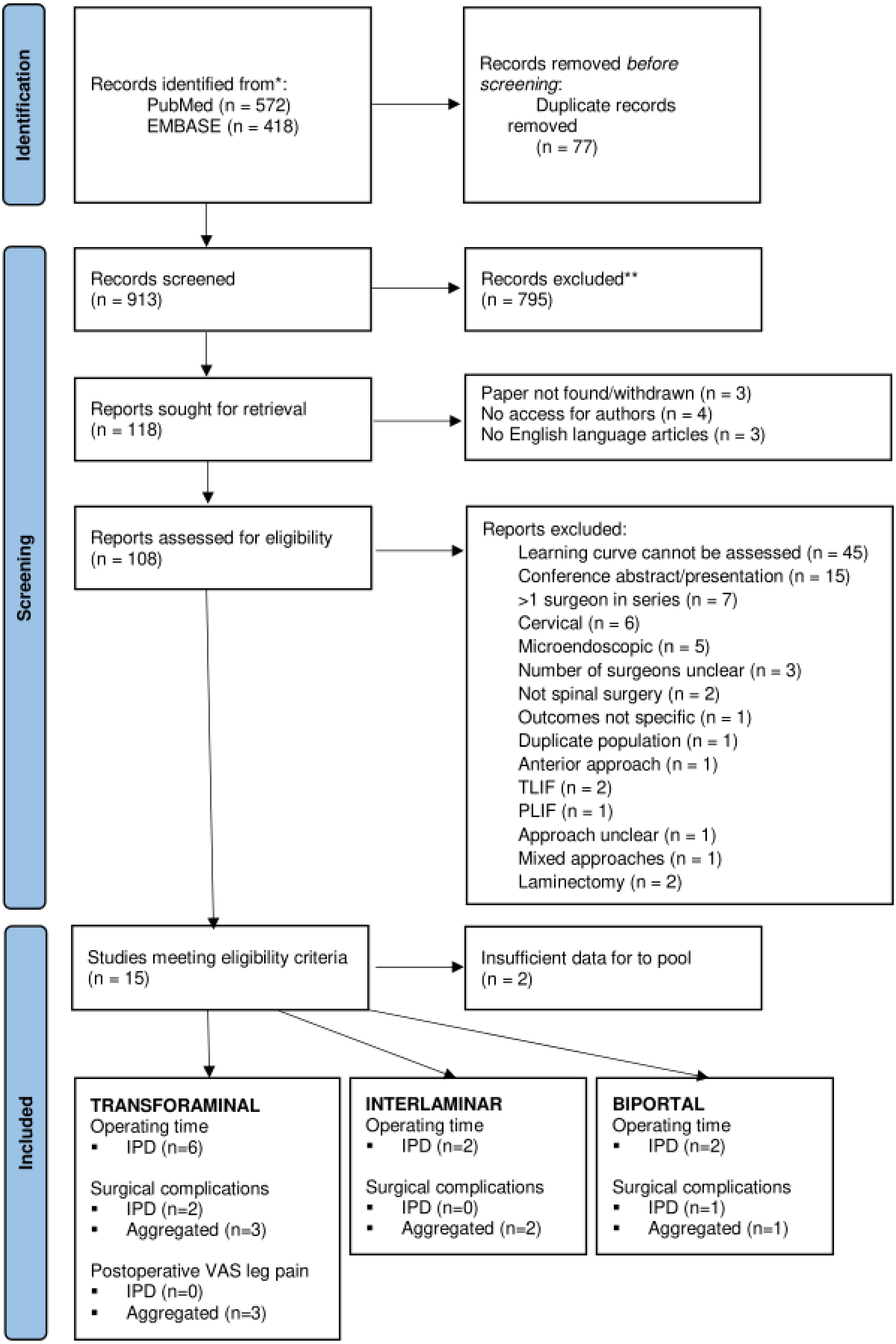
Identification
Records identified from*:
PubMed (n = 572)
EMBASE (n = 418)
Records removed *before screening*:
Duplicate records removed
(n = 77)
Screening
Records screened
(n = 913)
Records excluded**
(n = 795)
Reports sought for retrieval
(n = 118)
Paper not found/withdrawn (n = 3)
No access for authors (n = 4)
No English language articles (n = 3)
Reports assessed for eligibility
(n = 108)
Reports excluded:
Learning curve cannot be assessed (n = 45)
Conference abstract/presentation (n = 15)
>1 surgeon in series (n = 7)
Cervical (n = 6)
Microendoscopic (n = 5)
Number of surgeons unclear (n = 3)
Not spinal surgery (n = 2)
Outcomes not specific (n = 1)
Duplicate population (n = 1)
Anterior approach (n = 1)
TLIF (n = 2)
PLIF (n = 1)
Approach unclear (n = 1)
Mixed approaches (n = 1)
Laminectomy (n = 2)
Included
Studies meeting eligibility criteria
(n = 15)
Insufficient data for to pool
(n = 2)
TRANSFORAMINAL
Operating time
IPD (n=6)
Surgical complications
IPD (n=2)
Aggregated (n=3)
Postoperative VAS leg pain
IPD (n=0)
Aggregated (n=3)
INTERLAMINAR
Operating time
IPD (n=2)
Surgical complications
IPD (n=0)
Aggregated (n=2)
BIPORTAL
Operating time
IPD (n=2)
Surgical complications
IPD (n=1)
Aggregated (n=1)

Figure 2. Exponential decay

$$Y = C e^{-kt} + Asym$$

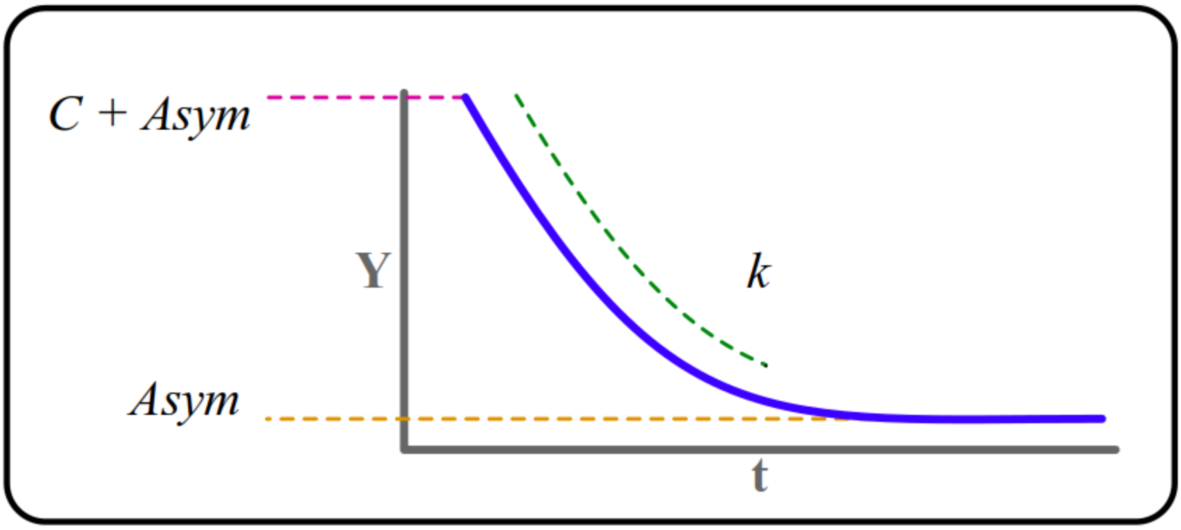

Supplementary Tables

Table 1. Summaries of included studies

REF	Author	Year	Mode	Approach	Procedure	No operations	Outcomes
[1]	**Lee**	**2009**	Uniportal Endoscopic	Transforaminal	Discectomy	51	Revision<br>Complications<br>Conversion
[2]	**Chaichankul**	**2012**	Uniportal Endoscopic	Transforaminal	Discectomy	50	VAS Leg pain
[3]	**Hsu**	**2013**	Uniportal Endoscopic	Transforaminal	Discectomy	33	Operating Time
[4]	**Ahn**	**2015**	Uniportal Endoscopic	Transforaminal	Discectomy	35	Operating Time<br>VAS Leg pain<br>Complications
[5]	**Wu**	**2016**	Uniportal Endoscopic	Transforaminal	Discectomy	120	Operating Time

REF	Author	Year	Mode	Approach	Procedure	No operations	Outcomes
[6]	**Joswig**	**2016**	Uniportal Endoscopic	Interlaminar	Discectomy<br>Discectomy	53<br>23	Conversion<br>Complications
[7]	**Fan**	**2017**	Uniportal Endoscopic	Transforaminal<br>Transforaminal	Discectomy<br>Discectomy	60<br>60	Operating Time
[8]	**Sun**	**2019**	Uniportal Endoscopic	Transforaminal	Discectomy	60	Macnab<br>Complications
[9]	**Zelenkov**	**2020**	Uniportal Endoscopic	Transforaminal: 28.1%<br>Interlaminar: 71.9%	Discectomy	57	Operating Time<br>Complications
[10]	**Son**	**2020**	Uniportal Endoscopic	Interlaminar	Discectomy	27	Operating Time
[11]	**Son**	**2021**	Uniportal Endoscopic	Transforaminal	Discectomy	48	Operating Time<br>Complications<br>Conversion

**REF**	**Author**	**Year**	**Mode**	**Approach**	**Procedure**	**No operations**	**Outcomes**
							Revision<br>VAS Leg pain
[12]	**Xu**	**2022**	Biportal Endoscopic	Interlaminar	Discectomy<br>Laminectomy	90<br>107	Operating Time<br>Complications
[13]	**Chen**	**2022**	Biportal Endoscopic	Interlaminar	Discectomy	97	Operating Time<br>VAS Leg pain<br>Macnab<br>Complications

Table 2. Quality Assessment

REF	Author	Year	Was the study question or objective clearly stated?	Was the study population clearly and fully described, including a case definition?	Were the cases consecutive?	Were the subjects comparable?	Was the intervention clearly described?	Were the outcome measures clearly defined, valid, reliable, and implemented consistently across all study participants?	Were the results well-described?
**1**	**Lee**	**2009**	Yes	Yes	Yes	Yes	Yes	Yes	No – grouped
**2**	**Chaichankul**	**2012**	Yes	Yes	Yes	Yes	Yes	Yes	No – grouped
**3**	**Hsu**	**2013**	Yes	Yes	Yes	Yes	Yes	Yes	No – most outcomes not on learning curve
**4**	**Ahn**	**2015**	Yes	Yes	Yes	Yes	Yes	Yes	Partly – most outcomes grouped
**5**	**Wu**	**2016**	Yes	Yes	No – separated by level	Yes	Yes	Yes	Yes
**6**	**Joswig**	**2016**	Yes	Yes	Yes	Yes	Yes	Yes	Partly – most outcomes are grouped
**7**	**Fan**	**2017**	Yes	Yes	Yes	Yes	Yes	Yes	No – grouped
**8**	**Sun**	**2019**	Yes	Yes	Yes	Yes	Yes	Yes	No – grouped
**9**	**Zelenkov**	**2020**	Yes	Yes	Yes	No – mixed TF and IL	Yes	No	Yes
**10**	**Son**	**2020**	Yes	Yes	Yes	Yes	Yes	Yes	Yes

**11**	**Son**	**2021**	Yes	Yes	Yes	Yes	Yes	Yes	Partly – most outcomes grouped
**12**	**Xu**	**2022**	Yes	Yes	Yes	Yes	Yes	Yes	No – either grouped, or raw data not presented, instead presented as CUSUM
**13**	**Chen**	**2022**	Yes	Yes	Yes	Yes	Yes	Yes	Partly – most outcomes grouped

References for included studies

[1] Lee DY, Lee S-H. Learning curve for percutaneous endoscopic lumbar discectomy. Neurol Med Chir (Tokyo) 2008;48:383–8; discussion 388-389. https://doi.org/10.2176/nmc.48.383.

[2] Chaichankul C, Poopitaya S, Tassanawipas W. The effect of learning curve on the results of percutaneous transforaminal endoscopic lumbar discectomy. J Med Assoc Thai 2012;95 Suppl 10:S206-212.

[3] Hsu H-T, Chang S-J, Yang SS, Chai CL. Learning curve of full-endoscopic lumbar discectomy. Eur Spine J 2013;22:727–33. https://doi.org/10.1007/s00586-012-2540-4.

[4] Ahn S-S, Kim S-H, Kim D-W. Learning Curve of Percutaneous Endoscopic Lumbar Discectomy Based on the Period (Early vs. Late) and Technique (in-and-out vs. in-and-out-and-in): A Retrospective Comparative Study. J Korean Neurosurg Soc 2015;58:539–46. https://doi.org/10.3340/jkns.2015.58.6.539.

[5] Wu X-B, Fan G-X, Gu X, Shen T-G, Guan X-F, Hu A-N, et al. Learning curves of percutaneous endoscopic lumbar discectomy in transforaminal approach at the L4/5 and L5/S1 levels: a comparative study. J Zhejiang Univ Sci B 2016;17:553–60. https://doi.org/10.1631/jzus.B1600002.

[6] Joswig H, Richter H, Haile SR, Hildebrandt G, Fournier J-Y. Introducing Interlaminar Full-Endoscopic Lumbar Diskectomy: A Critical Analysis of Complications, Recurrence Rates, and Outcome in View of Two Spinal Surgeons' Learning Curves. J Neurol Surg A Cent Eur Neurosurg 2016;77:406–15. https://doi.org/10.1055/s-0035-1570343.

[7] Fan G, Han R, Gu X, Zhang H, Guan X, Fan Y, et al. Navigation improves the learning curve of transforamimal percutaneous endoscopic lumbar discectomy. Int Orthop 2017;41:323–32. https://doi.org/10.1007/s00264-016-3281-5.

[8] Sun B, Shi C, Xu Z, Wu H, Zhang Y, Chen Y, et al. Learning Curve for Percutaneous Endoscopic Lumbar Diskectomy in Bi-needle Technique Using Cumulative Summation Test for Learning Curve. World Neurosurg 2019;129:e586–93. https://doi.org/10.1016/j.wneu.2019.05.227.

[9] Zelenkov P, Nazarov VV, Kisaryev S, Pimenova L, Zakirov BA, Goldberg M, et al. Learning Curve and Early Results of Interlaminar and Transforaminal Full-Endoscopic Resection of Lumbar Disc Herniations. Cureus 2020;12:e7157. https://doi.org/10.7759/cureus.7157.

[10] Son S, Ahn Y, Lee SG, Kim WK. Learning curve of percutaneous endoscopic interlaminar lumbar discectomy versus open lumbar microdiscectomy at the L5-S1 level. PLoS One 2020;15:e0236296. https://doi.org/10.1371/journal.pone.0236296.

[11] Son S, Ahn Y, Lee SG, Kim WK, Yoo BR, Jung JM, et al. Learning curve of percutaneous endoscopic transforaminal lumbar discectomy by a single surgeon. Medicine (Baltimore) 2021;100:e24346. https://doi.org/10.1097/MD.0000000000024346.

[12] Xu J, Wang D, Liu J, Zhu C, Bao J, Gao W, et al. Learning Curve and Complications of Unilateral Biportal Endoscopy: Cumulative Sum and Risk-Adjusted Cumulative Sum Analysis. Neurospine 2022;19:792–804. https://doi.org/10.14245/ns.2143116.558.

[13] Chen L, Zhu B, Zhong H-Z, Wang Y-G, Sun Y-S, Wang Q-F, et al. The Learning Curve of Unilateral Biportal Endoscopic (UBE) Spinal Surgery by CUSUM Analysis. Front Surg 2022;9:873691. https://doi.org/10.3389/fsurg.2022.873691.
